# Genetic parameters for growth, reproductive and maternal traits in a multibreed meat sheep population

**DOI:** 10.1590/S1415-47572009005000080

**Published:** 2009-12-01

**Authors:** Ana Maria Bezerra Oliveira Lôbo, Raimundo Nonato Braga Lôbo, Samuel Rezende Paiva, Sônia Maria Pinheiro de Oliveira, Olivardo Facó

**Affiliations:** 1 Departamento de Zootecnia, Universidade Federal do Ceará, Fortaleza, CEBrazil; 2 Embrapa Caprinos e Ovinos, Sobral, CEBrazil; 3 Embrapa Recursos Genéticos e Biotecnologia, Brasília, DFBrazil

**Keywords:** age at first lambing, genetic correlation, heritability, body weight, litter weight

## Abstract

The genetic parameters for growth, reproductive and maternal traits in a multibreed meat sheep population were estimated by applying the Average Information Restricted Maximum Likelihood method to an animal model. Data from a flock supported by the Programa de Melhoramento Genético de Caprinos e Ovinos de Corte (GENECOC) were used. The traits studied included birth weight (BW), weaning weight (WW), slaughter weight (SW), yearling weight (YW), weight gain from birth to weaning (GBW), weight gain from weaning to slaughter (GWS), weight gain from weaning to yearling (GWY), age at first lambing (AFL), lambing interval (LI), gestation length (GL), lambing date (LD - number of days between the start of breeding season and lambing), litter weight at birth (LWB) and litter weight at weaning (LWW). The direct heritabilities were 0.35, 0.81, 0.65, 0.49, 0.20, 0.15 and 0.39 for BW, WW, SW, YW, GBW, GWS and GWY, respectively, and 0.04, 0.06, 0.10, 0.05, 0.15 and 0.11 for AFL, LI, GL, LD, LWB and LWW, respectively. Positive genetic correlations were observed among body weights. In contrast, there was a negative genetic correlation between GBW and GWS (-0.49) and GBW and GWY (-0.56). Positive genetic correlations were observed between AFL and LI, LI and GL, and LWB and LWW. These results indicate a strong maternal influence in this herd and the presence of sufficient genetic variation to allow mass selection for growth traits. Additive effects were of little importance for reproductive traits, and other strategies are necessary to improve the performance of these animals.

## Introduction

The Brazilian meat sheep industry has expanded in recent years mainly through an increase in the number of farmers. However, performance indexes are lower than those required to guarantee efficiency and competitiveness compared with other animal sectors. Dickerson (1970) suggested that increasing the number of lambs marketed per ewe per year is an important measure to improve the efficiency of meat sheep production. However, in Brazil, local breeds show low productivity, which leads breeders to cross these with exotic breeds. Crossbreeding programs have been so widely used in the tropics that very few within-breed selection experiments have been done (Kosgey *et al.*, 2004). Because selection to enhance reproductive traits in sheep is rather slow, the crossbreeding of local breeds with highly prolific breeds is widespread in several countries, the aim being to increase lamb production by exploiting the additive and non-additive effects of genes (El Fadili and Leroy, 2001). The study of these aspects in Brazilian multibreed populations is necessary since most investigations have concentrated on performance analysis (Silva and Araújo, 2000; Silva, 2002; Barros *et al.*, 2004, 2005). Consequently, the genetic variation in these populations is unknown.

Hall *et al.* (1995) reported direct and maternal heritabilities for birth weight of 0.03 and 0.24, respectively, in crossbred animals, whereas Pitono and James (1995) stated that for a tropical breed the corresponding values for these heritabilities were 0.13 and 0.08, respectively. Genetic parameters for weaning weight, carcass traits and ovulation rates have been reported for crossbred and tropical breeds (Pollott *et al.*, 1994; Hall *et al.*, 1995; Pitono and James, 1995; Davis *et al.*, 1998). Most studies of genetic parameters in sheep have used purebred animals and have focused on body weights. Studies that have used crossbred and composite breeds include those of Waldron *et al.* (1992), Fossceco and Notter (1995), Jones *et al.* (1999), El Fadili and Leroy (2001) and Rosati *et al.* (2002).

Correct selection is one of the most important strategies to maximize production in animal breeding. However, the lack of estimates for the genetic parameters necessary to predict genetic gains is commonly cited as an obstacle in the design and implementation of conservation-based selective breeding programs in the tropics. As a result, there are few reports on successful selective breeding programs in this region (Gizaw *et al.*, 2007). Estimates of genetic parameters are necessary to determine the selection method to be used, to estimate the maximum genetic gain that can be achieved and to obtain correct estimates of breeding values. However, there have been few estimates of the genetic parameters for growth, reproductive and maternal traits in sheep in Brazil. Sousa *et al.* (1999, 2006) and Sarmento *et al.* (2006) described results obtained with the purebred Santa Inês, but there have been no studies with crossbred sheep.

According to Robinson *et al.* (1981), the use of models involving different genetic groups can account for all genetic influences, including non-linear and epistatic effects. However, additive genetic variance and heritability have been shown to be overestimated in an additive model with progeny groups in multibreed population. Van Der Werf and De Boer (1989) pointed out that crossbreeding parameters may be hard to estimate, particularly from field data, and that well-designed experiments are prerequisite for meaningful results. In their simulation study, the overestimation of additive variance and heritability was relatively small for levels of heterosis and recombination ≤ 5%. Rodriguez-Almeida *et al.* (1997) reported that the separation of estimates of the mean genetic effects on traits in multibreed populations of beef cattle into those attributable to the genetic make-up of the calf and those attributable to the genetic makeup of the dam required data from a variety of crosses. The authors considered that, in beef cattle, there are a limited number of breeds or crosses in any herd. Consequently, the estimation of direct and maternal breed genetic effects from field data sets may not be possible. The development of a system for evaluating crossbred records will require the incorporation of information from adequately designed crossbreeding experiments.

The lack of systematic record keeping by Brazilian sheep breeders means that there have been few studies of the genetic parameters related to reproductive and maternal traits in the Brazilian flock. This lack of information greatly hinders adequate development of the meat sheep industry in which the reproductive and maternal efficiencies of ewes must be constantly evaluated in order to ensure profitable production. The aim of this study was therefore to estimate the additive genetic parameters for growth, reproductive and maternal traits in meat sheep of a multibreed population raised in Brazil.

## Materials and Methods

The data bank analyzed contained 12 years (1996-2007) of information from a flock maintained by Gaasa Agropecuária Ltda. and supported by the Programa de Melhoramento Genético de Caprinos e Ovinos de Corte (GENECOC) of Embrapa Caprinos e Ovinos. This herd is located at Inhumas in the state of Goiás (altitude 770 m, 16° 21' 28” S, 49° 29' 45” W) with a tropical semi-humid climate.

The sheep underwent standard sanitary care and were vaccinated with Poli-Star^®^ (against botulism, enterotoxemia, gangrene and symptomatic carbuncle) at 50 and 80 days of age; all of the animals were vaccinated annually in March. Treatment for eimeriosis was done twice a year (March and October). Fecal egg counts and the Famacha method were used to control gastrointestinal nematodes. Footbaths (50 g of copper sulfate in 60 mL of 40% formaldehyde) were used during the rainy season.

The breeding season was year round with ewes grouped by lots. The lambs were weaned at 60 days, confined, and fed with maize silage and corn and soy bran meal containing 21% crude protein (CP); they were slaughtered at 120-150 days. After weaning the lambs, the ewes underwent a 30-day breeding season in the presence of vasectomized rams. Ewes and rams were fed with Tifton 85 pasture, silage and meal containing 15% CP.

To understand the flock formation, initially purebred Santa Inês, Poll Dorset, Hampshire Down, Suffolk, Île de France, Brazilian Somali and Texel rams were acquired with Santa Inês, Poll Dorset, Morada Nova, Brazilian Somali, Santa Inês x Morada Nova and Santa Inês x Brazilian Somali dams. Later, purebred Dorper, Primera and East Friesian rams were acquired. The matings were controlled but not designed. All rams of all breeds had the same opportunity to mate ewes of all genetic groups (purebred and crossbred). As a result, within a few years the flock was a mixture of crossbred dams with a varied contribution from the different breeds. Only the rams and some Santa Inês, Poll Dorset and Brazilian Somali dams were purebred.

Data used in this study were obtained several years after formation of the flock, and already contained of the all breeds indicated above. However, many genetic groups were excluded from analysis because of insufficient information. The rams used included purebred Santa Inês (36), Poll Dorset (16), Hampshire Down (5), East Friesian (4), Dorper (4), Suffolk (3), Île de France (2), Brazilian Somali (2), Texel (1) and Primera (1). The dams used included purebred Santa Inês, Poll Dorset, Brazilian Somali and crossbreeds involving all of the breeds indicated above. Table 1 shows the genetic groups analyzed.

The growth traits analyzed included birth weight (BW), weaning weight (WW), slaughter weight (SW), yearling weight (YW), weight gain from birth to weaning (GBW), weight gain from weaning to slaughter (GWS) and weight gain from weaning to yearling (GWY). The WW of lambs > 30 days old but weighing < 5 kg were excluded. Likewise, the SW of animals with a slaughter age > 365 days were also excluded. All weights from animals 330-395 days old were considered as YW. The YW of lambs > 20 kg were excluded.

The reproductive and maternal traits analyzed included age at first lambing (AFL), lambing interval (LI), gestation length (GL), lambing date (LD; number of days between the start of breeding season and lambing), litter weight at birth (LWB) and litter weight at weaning (LWW). Data for animals with an AFL > 800 days and an LI > 550 days were excluded. Lambing orders from 1 to 8 were analyzed.

The MIXED procedure of SAS (SAS Institute Inc., 1996) was used to define fixed effects in the analysis. For each trait, many linear models were evaluated, with the use of contemporary groups or effects being analyzed individually. The logarithm of the Restricted Maximum Likelihood, Aikaikes's Information Criteria and Schwarz's Bayesian Information Criteria were the criteria for choice of the best fit.

For growth traits, contemporary groups (CG) were formed by grouping animals born in the same year-season and with the same sex, genetic group and birth type (single, twin, triplet). Only CGs with a minimum of five animals were considered. The birth seasons were: season 1 - January, February and March, season 2 - April, May and June, season 3 - July, August and September, and season 4 - October, November and December.

For reproductive and maternal traits, two CGs were created: the first for AFL and the second for other traits. For AFL, the CG consisted of animals born in the same year-season, of the same genetic group, birth type (single, twin, triplet) and interaction of birth type with lamb sex (1 - one male lamb, 2 - one female lamb, 3 - two male lambs, 4 - two females lambs, 5 - one male lamb and one female lamb or 6 - more than two lambs, independent of sex). For the other traits, the CGs consisted of animals of the same genetic group, with lambing in the same year-season, the same birth type (1 to 6, as defined above) and the same lambing order. Only CGs with a minimum of five animals were considered.

After preliminary analysis and based on criteria used to determine the best fit, the following fixed models were used, depending on the trait:

BW - contemporary group and age class of the dam at lambing (1 to 8 years);

WW and GBW - contemporary group, age class of the dam at lambing and lamb age (days) at weaning as linear and quadratic covariates;

SW and GWS - contemporary group and animal age (days) at slaughter as the covariate (linear and quadratic);

YW and GWY - contemporary group and animal age (days) on the date the animal was weighed as the covariate (linear and quadratic);

AFL, LI, GL, LD, TBW and TWW - contemporary groups.

The (co)variances and genetic parameters were estimated by the Average Information Restricted Maximum Likelihood method (AI-REML) using the software WOMBAT (Meyer, 2006), with single or multiple trait animal models. WOMBAT assesses whether an analysis has converged, based on the following criteria: 1) a change in log L of < 5x10^-4^, 2) a change in parameters of < 10^-8^ and 3) a gradient vector norm < 10^-3^.

The relationship matrix included 16,808 animals. Of these, 75 were rams and 4,272 were ewes, 13,124 animals had a complete pedigree (sire and dam known) and 1,251 were from the basal flock; 224 animals had an unknown sire. There was an average of 156 lambs per ram and 901 lambs per ram breed.

The general model for growth traits was:



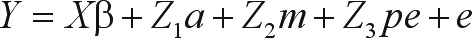


where *Y* is a (N x 1) vector of observations, β is the vector of fixed effects related to the incidence matrix *X*, a is the vector of direct genetic effects related to the incidence matrix *Z*_1_, *m* is the vector of maternal genetic effects related to the incidence matrix *Z*_2_, pe is the vector of permanent environmental maternal effects related to the incidence matrix *Z*_3,_ and *e* is the vector of random residuals.

The fixed effects have already been described previously. The general model presented above was used for BW, WW and GBW. The components *Z*_2_*m* and *Z*_3_*pe* were not included in the analysis of SW, YW, GWS and GWY. BW, WW and SW were analyzed in a multiple trait model. YW was analyzed in a multiple trait model with BW and WW. GBW was analyzed with GWS in one analysis and with GWY in another analysis.

Using the same general model described previously for all reproductive and maternal traits, the *Z*_2_*m* component was not considered and the *Z*_3_*pe* component refers to permanent environmental effects on the animal and was included only for traits measured many times on the same animal (LI, GL, LD, TBW and TWW). These two components were not included for AFL. AFL, LI and GL were analyzed in a multiple trait model, as were TBW and TWW. LD was analyzed in a single trait model.

The crossbreeding in this population was completely random, *i.e.*, there was no specific design in the breeding pattern used. As an initial simplification in the data analysis, the average effect (additive and non-additive) of the genetic groups was considered to be fixed.

## Results

The number of observations and the means for the traits analyzed are shown in Table 2. The means for WW and SW were 15.52 kg and 31.96 kg, respectively. In this flock, the average ages at weaning and slaughter were 55.68 ± 12.41 days and 128.74 ± 44.97 days, respectively, indicating a high potential for precocity under the feeding conditions used here.

The estimates of (co)variance, Log L value, heritabilities and genetic correlations for a multiple trait analysis for BW, WW and SW are summarized in Table 3. Negative genetic covariances were observed between direct and maternal effects for BW and WW. The direct heritabilities for BW, WW and SW were moderate to high (0.35-0.81). The maternal heritability for WW (0.21) was higher than for BW (0.17), indicating moderate genetic maternal variability in the flock that could be used to select for maternal ability. The genetic correlations among these traits were high (0.70-0.92).

Table 4 shows the (co)variances, Log L value, heritabilities and genetic correlations for multiple trait analysis with BW, WW and YW. As with BW, WW and SW, there were negative genetic covariances between genetic and maternal effects for BW and WW. The direct heritabilities ranged from moderate to high, indicating a potential for the selection of these traits in this flock. Heritability for BW was higher than in the previous analysis (0.42 *vs.* 0.35) but was lower for WW (0.60 *vs.* 0.81). The genetic correlations were high, with a maximum of 0.89 between BW and WW, as also observed in the analysis with BW, WW and SW. Comparison of Tables 3  and 4 shows that there were similarities between the two multiple trait analyses.

The (co)variances, Log L value, heritabilities and genetic correlations for multiple trait analysis with GBW and GWS are summarized in Table 5. The results for the analysis using GBW and GWY are shown in Table 6. Direct heritabilities were 0.15 and 0.39 for GWS and GWY, respectively. Direct and maternal heritabilities for GBW ranged from 0.20 to 0.36 and from 0.06 to 0.11, respectively, being lowest in the analysis with GBW and GWS. GBW was negatively correlated with posterior weight gains (-0.49 with GWS and -0.56 with GWY).

Table 7 shows the estimates of (co)variances, Log L value, heritabilities and genetic correlations for multiple trait analysis with AFL, LI and GL. The heritabilities were low, ranging from 0.04 to 0.10. The genetic correlation between AFL and GL was negative (-0.33), but was positive between AFL and LI and between LI and GL (0.19 each). However, the errors associated with these correlations were very high.

The (co)variances, Log L value, heritabilities and genetic correlation for the maternal traits LWB and LWW are shown in Table 8. The was a high, positive genetic correlation between these traits (0.86) while the heritabilities were low (0.15 for LWB and 0.11 for LWW).

Table 9 shows the estimated parameters for LD. The heritability for this trait was low (0.05), indicating a strong influence of environmental and non-additive genetic effects on the traits examined. This estimate was similar to that for the lambing interval.

## Discussion

Direct additive variances and heritabilities were higher than maternal genetic variances and heritabilities for all growth traits. In general, direct heritabilities tend to be higher than maternal heritabilities for early growth traits (Hassen *et al.*, 2003). Sarmento *et al.* (2006) observed heritabilities of 0.20 and 0.001 for birth weight and weaning weight, respectively, at 112 days of age in purebred Santa Inês sheep. Their estimate for BW was similar to that found here. This breed has a larger contribution in the population studied here (Table 1), which may explain this similarity. Tosh and Kemp (1994) estimated direct heritabilities of 0.39 and 0.22 for BW and WW in Hampshire Down purebred sheep. Lower direct heritabilities for BW and WW, respectively, have been reported by others, *e.g.*, 0.12 and 0.21 for Poll Dorset sheep (Tosh and Kemp, 1994), 0.09 and 0.09 for composite sheep (Mousa *et al.*, 1999), 0.13 and 0.04 for Santa Inês sheep (Sousa *et al.*, 1999) and 0.24 and 0.19 for dual purpose (wool and meat) crossbred sheep (Hall *et al.*, 1995). Genetic parameters depend on the historical formation, selective forces and environmental aspects of a population, which partly explains differences among the results of different studies. However, it is possible that the values reported here were overestimated by non-additive effects.

Safari *et al.* (2005) reviewed the genetic parameters for growth, carcass and reproductive traits compiled from 165 studies in sheep published from 1992 to 2003, with estimates generally derived from mixed model REML procedures, in addition to some Bayesian estimates. The weighted means for direct heritability associated with birth, weaning and post-weaning weights and daily gain were 0.15 ± 0.02, 0.18 ± 0.04, 0.21 ± 0.01 and 0.17 ± 0.01, respectively, for meat breeds. The estimates described here were greater than the weighted means reported by these authors, which suggests that bias in our analysis resulted in overestimation of the parameters. According to Van Der Werf and De Boer (1989), additive genetic variance and heritability are overestimated when data from crossbred animals are analyzed by additive model without non-additive effects.

The maternal heritabilities for BW and WW were different from those of Sousa *et al.* (1999) for a purebred Santa Inês flock (0.12 for BW and 0.10 for WW) and of Mousa *et al.* (1999) for a composite breed, but were similar to those of Maria *et al.* (1993) for Romanov sheep. Tosh and Kemp (1994) reported maternal heritabilities of 0.22, 0.31 and 0.13 for BW and 0.14, 0.19 and 0.06 for weight at 50 days of age in Hampshire Down, Poll Dorset and Romanov sheep, respectively. In dual purpose crossbred sheep, Hall *et al.* (1995) reported maternal heritabilities of 0.08 for BW and 0.05 for WW. As shown here, the maternal effect was strong for BW and WW, indicating the importance of maternal ability in crossbred females in this population. Differences in the maternal effects among breeds have been attributed to variations in milk production (Meyer *et al.*, 1994; Tosh and Kemp, 1994).

The (co)variance and correlation estimates between animal and maternal genetic effects were generally negative for all traits. Sarmento *et al.* (2006) reported genetic correlations (r_am_) of -0.47 and -0.24 between the direct and maternal effects for BW and WW, respectively, in purebred Santa Inês. These values were similar to those observed here, possibly because this breed provided the largest contribution to the flock in our study. Sousa *et al.* (1999), who also studied Santa Inês sheep, reported correlations of -0.15 and -0.10 between direct and maternal effects for birth weight and weaning weight, respectively, at 112 days of age. Hassen *et al.* (2003) likewise reported antagonism between direct and maternal effects: from -0.48 to -0.23 for BW and from -0.69 to -0.57 for WW. Tosh and Kemp (1994) observed r_am_ of -0.56 and -0.35 for BW in Hampshire Down and Poll Dorset sheep, respectively. Janssens *et al.* (2000) reported an r_am_ of 0.03 for Belgian Texel sheep and Maniatis and Pollott (2002) reported a value of -0.64 for Suffolk sheep. In contrast, there have been no estimates for this correlation in multibreed sheep populations. The value estimated here suggests that it may be difficult for breeders to select for both aspects. Selection for direct effects allows a reduction in maternal effects and vice-versa.

There is a lack of information on the genetic parameters for YW in Brazilian flocks. Matika *et al.* (2003) observed a direct heritability of 0.26 for weight at 12 months. Miraei-Ashtiani *et al.* (2007) cited a heritability of 0.10 ± 0.05 for yearling weight of Sangsari sheep in Iran, and Mokhtari *et al.* (2008) estimated the heritability of YW to be 0.15 in Kermani sheep. These values were lower than observed here, suggesting that we overestimated this parameter, probably because of non-addictive effects.

The high correlations between BW and WW (0.83), BW and SW (0.70) and WW and SW (0.92) indicated the possibility of a correlated response with the selection for a given trait. The greater heritability of SW and the high correlation between WW and SW suggested that animals may be selected at an early age.

Genetic correlations ranging from -0.33 to 0.81 have been reported between BW and WW in different breeds and at different ages (Lewer *et al.*, 1994; Vaez Torshizi *et al.*, 1996; Analla *et al.*, 1997; Yazdi *et al.*, 1997; Mousa *et al.*, 1999; Neser *et al.*, 2001; Wuliji *et al.*, 2001; Boujenane and Kansari, 2002; Duguma *et al.*, 2002; Hanford *et al.*, 2002; Simm *et al.*, 2002). Correlations between BW and weight at 90 days of age (0.56) and post-weaning weight at 120 days of age (0.44) were reported by Nasholm and Danell (1996) in Swedish finewool sheep, and between BW and weight at 174 days of age (0.48) by Analla *et al.* (1997) in Merino sheep. High genetic correlations (0.83-0.98) between WW and post-weaning weights have also been reported (Nagy *et al.*, 1999; Wuliji *et al.*, 2001; Yazdi *et al.*, 1997; Snyman *et al.*, 1998). Miraei-Ashtiani *et al.* (2007) reported a genetic correlation of 0.43 between WW and YW, and Gizaw *et al.* (2007) estimated this correlation to be 0.69 ± 0.01. These estimates were lower than described here, probably because we overestimated these values as a result of non-additive effects.

A knowledge of the magnitude and directions of the correlations among traits is important for establishing efficient selection strategies. As expected, the correlation between WW and YW was greater than between BW and YW. Nevertheless, in both cases, the correlations indicated that selection for one trait would positively affect the response to the other.

An understanding of the additive genetic variation for weight gain is extremely important for breeding programs. The direct (0.20) and maternal (0.06) heritabilities for GBW (Table 5) were similar to those reported by Maria *et al.* (1993) for Romanov sheep. Matika *et al.* (2003) reported direct and maternal heritabilities and correlation between direct and maternal effects of 0.17, 0.04 and -0.08, respectively, for the pre-weaning average daily weight gain in Sabi sheep. Hagger (1998) observed a correlation of -0.45 between the direct and maternal effects for this same trait. The direct heritability estimated here for GWY was similar to that reported by Mousa *et al.* (1999) for post-weaning average daily weight gain in a composite terminal sire breed.

The negative correlations between GBW and GWS/GWY may reflect the limited number of observations for these parameters, partly because of the way the data were distributed. The animals in this flock were slaughtered between 120 and 150 days of age (128 on average), with only the males and females selected for reproduction reaching one year of age. Consequently, birth-to-weaning data were distributed into the weaning-to-slaughter and weaning-to-yearling groups. In addition, some lambs did not reach weaning. These negative correlations may also reflect differences in the animals responses to pre- and post-weaning environmental conditions, such as occurs in genotype *vs.* environmental interactions. In the pre-weaning period, the animals were maintained in confined conditions, whereas after weaning they had access to pasture and food supplementation.

AFL and LI were strongly influenced by environmental effects, whereas GL showed little inter-individual variation. Hence, the low heritabilities estimated for these traits were expected. A heritability of 0.27 for LI was estimated by McManus and Miranda (1998), but there was a high standard error (0.29). The low estimates observed here for reproductive traits did not mean that there was no possibility for genetic improvement, but rather that the expected genetic gain was low if selection for these traits was also low.

According Rosati *et al.* (2002), a low heritability for reproductive traits probably reflects a proportionally greater influence of environmental effects, as well as a low genetic variability for fertility, litter size, lamb survival, lambing frequency and other reproductive traits (Turner and Young, 1969). Heritability for LWB was similar to that reported by McManus and Miranda (1998), but their estimate of heritability for LWW differed from that observed here. Rosati *et al.* (2002) reported heritabilities of 0.40 and 0.17 for total litter weight at birth and total litter weight at weaning for purebred, composite and crossbred sheep based on an additive model in which the ewe breed effect was fixed (as done here). These authors commented on the high values they obtained for these traits and pointed out that heritability estimates may be influenced by other factors not considered in the model; non-additive effects were not considered and their estimates were generally higher than observed here. The large positive genetic correlation between LWB and LWW suggests that productivity can also be selected based on LWW.

The similarity between the values for LD and LI suggests that these traits had essentially the same characteristics, possibly because of the reproductive management of the flock. The exposure of ewes to mating seasons meant that LI was probably not a good trait for selection because of possible bias. In this case, LD would probably be more efficient for selecting fertility because there would be no breeder-introduced bias, *i.e.*, the ewes would be unable to express their full reproductive potential because the mating season was limited to a particular period. However, since in this flock the mating season was essentially continuous the year round, all of the ewes had an opportunity to mate at eight-month intervals.

In conclusion, the results of this study show that there is strong maternal influence in this herd and the presence of sufficient genetic variation to allow mass selection for growth traits. Additive effects were of little importance for reproductive traits, and other strategies are necessary to improve the performance of these animals. However, the estimates presented here should be interpreted cautiously because of possible bias introduced by the absence of non-additive effects in the model used here. This model was chosen primarily because the lack of information on genetic parameters in Brazilian sheep precluded the choice of a more adequate one. Consequently, it is possible that the additive variance and heritability for some traits were overestimated. Nevertheless, the results described here provide a starting point for more detailed studies on the genetic parameters of multibreed sheep in Brazil.

## Figures and Tables

**Table 1:** Genetic groups analyzed in this work and the number of observations (N).

Genetic groups	N
1/2 Dorper + 1/2 Santa Inês	275
1/2 Dorper + 1/2 Brazilian Somali	202
1/2 Dorper + 1/4 Île de France + 1/4 Santa Inês	36
1/2 Dorper + 1/4 Poll Dorset + 1/4 Santa Inês	198
1/2 Dorper + 1/4 Suffolk + 1/4 Santa Inês	42
1/2 Dorper + 1/4 Texel + 1/4 Santa Inês	103
1/2 East Friesian + 1/2 Santa Inês	61
1/2 Hampshire Down + 1/2 Santa Inês	128
1/2 Île de France + 1/2 Santa Inês	125
1/2 Île de France + 1/4 Poll Dorset + 1/4 Santa Inês	24
1/2 Poll Dorset + 1/2 Santa Inês	681
1/2 Poll Dorset + 1/2 Brazilian Somali	43
1/2 Poll Dorset + 1/4 Dorper + 1/4 Santa Inês	25
1/2 Primera + 1/2 Santa Inês	44
1/2 Primera + 1/4 Poll Dorset + 1/4 Santa Inês	26
1/2 Santa Inês + 1/2 Brazilian Somali	145
1/2 Suffolk + 1/2 Santa Inês	65
1/2 Texel + 1/2 Santa Inês	102
3/4 Dorper + 1/4 Santa Inês	223
3/4 Dorper + 1/4 Brazilian Somali	115
3/4 Dorper + 1/8 Île de France + 1/8 Santa Inês	35
3/4 Dorper + 1/8 Poll Dorset + 1/8 Santa Inês	80
3/4 Poll Dorset + 1/4 Santa Inês	315
3/4 Santa Inês + 1/4 Hampshire Down	23
3/4 Santa Inês + 1/4 Île de France	28
3/4 Santa Inês + 1/4 Poll Dorset	131
3/4 Santa Inês + 1/4 Brazilian Somali	62
3/4 Santa Inês + 1/4 Suffolk	36
3/4 Santa Inês + 1/4 Texel	22
7/8 Santa Inês + 1/8 Poll Dorset	42
Poll Dorset	170
Santa Inês	8212
Brazilian Somali	124

**Table 2:** Number of observations (N), means and standard deviations (SD) for the traits analyzed in this work.

Trait	N	Mean ± SD
Birth weight (kg)	11,943	3.84 ± 0.86
Weaning weight (kg)	10,043	15.52 ± 3.99
Slaughter weight (kg)	1,542	31.96 ± 6.22
Yearling weight (kg)	1,208	41.15 ± 7.64
Weight gain from birth to weaning (kg/day)	10,043	0.213 ± 0.063
Weight gain from weaning to slaughter (kg/day)	1,490	0.234 ± 0.085
Weight gain from weaning to yearling (kg/day)	1,177	0.087 ± 0.024
Age at first lambing (days)	2,154	532.71 ± 90.20
Lambing interval (days)	4,600	267.23 ± 56.87
Gestation length (days)	6,930	150.93 ± 3.20
Lambing date (days)	1,437	164.72 ± 9.04
Litter weight at birth (kg)	7,022	4.95 ± 1.55
Litter weight at weaning (kg)	6,217	18.34 ± 6.73

**Table 3:**
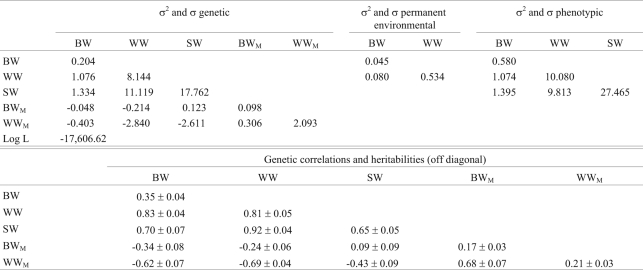
Estimates of (co)variances (kg^2^), heritabilities and genetic correlations for birth weight (BW), weaning weight (WW) and slaughter weight (SW) in the multiple trait model.

σ^2^ = variances and σ = covariances. BW = genetic direct effect for birth weight, BW_M_ = genetic maternal effect for birth weight, Log L = logarithm of the Likelihood function, SW = genetic direct effect for slaughter weight, WW = genetic direct effect for weaning weight and WW_M_ = genetic maternal effect for weaning weight.

**Table 4:**
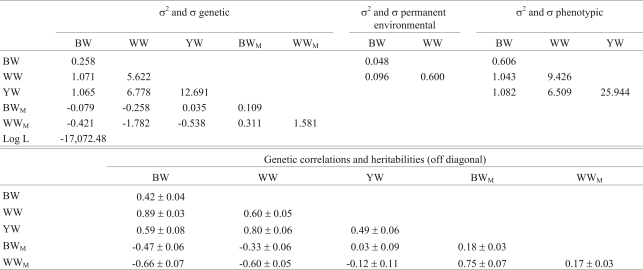
Estimates of (co)variances (kg^2^), heritabilities and genetic correlations for birth weight (BW), weaning weight (WW) and yearling weight (YW) in the multiple trait model.

σ^2^ = variances and σ = covariances. BW = genetic direct effect for birth weight, BW_M_ = genetic maternal effect for birth weight, Log L = logarithm of the Likelihood function, WW = genetic direct effect for weaning weight, WW_M_ = genetic maternal effect for weaning weight and YW = genetic direct effect for yearling weight.

**Table 5:**
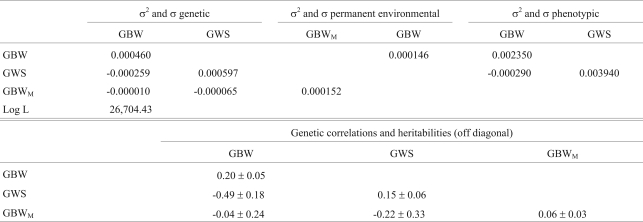
Estimates of (co)variances (kg/day^2^), heritabilities and genetic correlations for weight gain from birth to weaning (GBW) and weight gain from weaning to slaughter (GWS) in the multiple trait model.

σ^2^ = variances and σ = covariances. GBW = genetic direct effect for weight gain from birth to weaning, GBW_M_ = genetic maternal effect for weight gain from birth to weaning, GWS = genetic direct effect for weight gain from weaning to slaughter and Log L = logarithm of the Likelihood function.

**Table 6:**
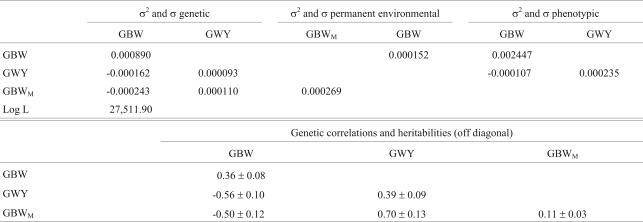
Estimates of (co)variances (kg/day^2^), heritabilities and genetic correlations for weight gain from birth to weaning (GBW) and weight gain from weaning to yearling (GWY) in the multiple trait model.

σ^2^ = variances and σ = covariances. GBW = genetic direct effect for weight gain from birth to weaning, GBW_M_ = genetic maternal effect for weight gain from birth to weaning, GWY = genetic direct effect for weight gain from weaning to yearling and Log L = logarithm of the Likelihood function.

**Table 7:**
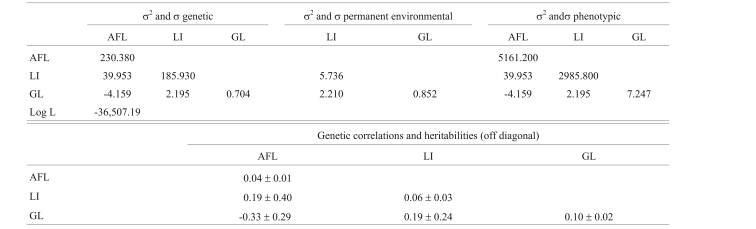
Estimates of (co)variances (day^2^), heritabilities and genetic correlations for age at first lambing (AFL), lambing interval (LI) and gestation length (GL) in the multiple trait model.

σ^2^ = variances and σ = covariances. Log L = logarithm of the Likelihood function.

**Table 8:** Estimates of (co)variances (kg^2^), heritabilities (h^2^) and genetic correlation for litter weight at birth (LWB) and litter weight at weaning (LWW) in the multiple trait model.

	σ^2^ and σ genetic			σ^2^ and σ permanent environmental			σ^2^ andσ phenotypic			
	LWB	LWW		LWB	LWW		LWB	LWW	h^2^	r_g_
LWB	0.144			0.053			0.947		0.15 ± 0.02	0.86 ± 0.08
LWW	0.604	3.431		0.315	1.857		0.920	31.239	0.11 ± 0.02	
Log L	-16,105.35									

σ^2^ = variances and σ = covariances. h^2^ = heritability, r_g_ = genetic correlation and Log L = logarithm of the Likelihood function.

**Table 9:** Estimates of variances (σ^2^; day^2^), heritability (h^2^) and logarithm of the Likelihood function (-2Log L) for lambing date (LD) in the multiple trait model.

Parameters	LD
σ^2^ genetic	3.3072
σ^2^ permanent environmental	0.2271
σ^2^ phenotypic	68.7890
h^2^	0.05 ± 0.05
Log L	-3,263.23
